# Exploring the expression and potential function of follicle stimulating hormone receptor in extragonadal cells related to abdominal aortic aneurysm

**DOI:** 10.1371/journal.pone.0285607

**Published:** 2023-05-25

**Authors:** V. N. Tedjawirja, A. Mieremet, K. B. Rombouts, C. Yap, A. E. Neele, B. H. Northoff, H. J. Chen, M. Vos, D. Klaver, K. K. Yeung, R. Balm, V. de Waard

**Affiliations:** 1 Department of Surgery, Amsterdam UMC, Location University of Amsterdam, Amsterdam Cardiovascular Sciences, Amsterdam, The Netherlands; 2 Department of Medical Biochemistry, Amsterdam UMC, University of Amsterdam, Amsterdam Cardiovascular Sciences, Amsterdam, The Netherlands; 3 Department of Surgery and Physiology, Amsterdam UMC, Location Vrije Universiteit Amsterdam, Amsterdam Cardiovascular Sciences, Amsterdam, The Netherlands; 4 Institute of Laboratory Medicine, Ludwig Maximilians University Munich, Munich, Germany; Yanbian University Hospital, CHINA

## Abstract

**Introduction:**

Follicle stimulating hormone (FSH) is identified to play a role in postmenopausal disease and hypothesized to affect abdominal aortic aneurysm (AAA) onset/progression in postmenopausal women. We aimed to detect *FSHR* gene expression in AAA tissue and cell types involved in AAA formation.

**Methods:**

FSH stimulation of human umbilical cord endothelial cells (HUVECs), smooth muscle cells (HUCs) and PMA-differentiated macrophages to assess gene expression of *FSHR* and various markers. Human macrophages activated with various stimuli were assessed for *FSHR* gene expression. AAA dataset, AAA tissue samples and AAA-derived smooth muscle cells (SMC) obtained from elderly female donors were assessed for *FSHR* gene expression. AAA-SMCs were stimulated with FSH to assess its effect on gene expression. Lastly, oxidized low-density-lipoprotein (ox-LDL) uptake and abundance of cell surface protein markers were assessed by flow cytometry after FSH stimulation of human monocytes.

**Results:**

FSH stimulation showed similar levels of gene expression in HUVECs and HUCs. Only *ACTA2* was downregulated in HUCs. In PMA-differentiated macrophages, gene expression of inflammation markers was unchanged after FSH stimulation. *FSHR* gene expression was found to be low in the AAA datasets. Female AAA-SMCs show occasional *FSHR* gene expression at a very low level, yet stimulation with FSH did not affect gene expression of SMC- or inflammation markers. FSH stimulation did not impact ox-LDL uptake or alter cell surface protein expression in monocytes. While *FSHR* gene expression was detected in human testis tissue, it was below quantification level in all other investigated cell types, even upon activation of macrophages with various stimuli.

**Conclusion:**

Despite previous reports, we did not detect *FSHR* gene expression in various extragonadal cell types, except in occasional female AAA-SMCs. No clear effect on cell activation was observed upon FSH stimulation in any cell type. Our data suggest that a direct effect of FSH in AAA-related extragonadal cells is unlikely to influence AAA.

## Introduction

An abdominal aortic aneurysm (AAA) is a dilatation of the abdominal aorta with a diameter ≥ 30mm and can be potentially fatal once rupture occurs [1]. Women with AAA have a higher risk of morbidity than men. This is reflected by increased AAA growth rates [2,3], which are currently unexplained. It has been suggested that women are protected against AAA by endogenous estrogen [4]–a protective effect that diminishes after the menopause. If estrogen were solely responsible for AAA protection in postmenopausal women, then hormonal replacement therapy would seemingly inhibit AAA progression. Yet, the effect of hormonal replacement therapy on AAA in postmenopausal women is inconclusive [5–8], and as such, perhaps the involvement of another factor could be considered. Recently, the chronically elevated levels of follicle stimulating hormone (FSH) present during the menopausal transition have been identified as playing a role in postmenopausal osteoporosis and cardiovascular disease [9], rather than the decline in the protective effect of estrogen during and/or after the menopause. In prior work we hypothesized that FSH may play a direct and/or indirect role in AAA onset and/or progression in postmenopausal women [10]. We explore the potential direct effects of this in the current study.

Prior research has reported the expression of the FSH receptor (FSHR) on endothelial cells (EC) and monocytes [11–14]. In human umbilical vein EC (HUVECs), FSH was reported to promote monocyte adhesion to vascular EC through upregulation of adhesive molecules (VCAM-1, ICAM-1and E-selectin) and also angiogenesis [11,13]. FSH stimulation was shown to increase TNF-α production in monocytes [12]. These cell types are also present in the aortic wall and therefore we aimed to explore *FSHR* gene expression and the effect of FSH in these cells. Smooth muscle cells (SMCs) lie adjacent to the endothelial cells (EC), are the most abundant cell type in the aorta, and are severely affected in AAA. As such, we also investigated the SMCs of human umbilical cord arteries (HUCs) and those derived from female AAA tissue. Since inflammation with vascular degradation is considered to be involved in all stages of AAA [15], in the current report we place the emphasis on monocytes/macrophages. Thus, the current study aims to detect *FSHR* gene expression in the aforementioned cell types and in AAA tissue in order to explore if FSH stimulation would affect enhanced AAA progression in postmenopausal women.

## Materials and methods

### Endothelial cells and smooth muscle cells

ECs from the human umbilical vein are most widely used as model cell and have been reported to express the FSHR and respond to FSH. Thus, this cell type was first chosen on which to perform experiments with FSH stimulation. Since SMCs are also present in the aorta, those of human umbilical arteries were taken as a model SMC (HUCs). EC and SMC were derived from the umbilical cord vessels provided by our department of Gynecology and Obstetrics. The umbilical cords are considered residual material and were used anonymously for scientific research purposes. HUVECs were isolated by cannulation of the vein with a trypsin solution (Sigma) and cultured for experiments to confluency on 0.1% gelatin-coated (Merck) wells in Endothelial Cell Growth Basal Medium-2 (EBM-2) (Promocell) with 2% fetal calf serum (FCS, Gibco) and 1% penicillin and streptomycin (P/S, Gibco). HUCs were isolated from explant cultures of the human umbilical cord arteries and were cultured in 0.1% gelatin coated flasks using DMEM/F12 (Gibco Dulbecco’s Modified Eagle Medium: Nutrient mixture F-12) with 10% FCS and 1% P/S. After reaching confluency, the cell culture medium was changed with DMEM F12 with P/S and 0.5% FCS to make the cells quiescent.

HUVECs and HUCs were treated with FSH (Follitropin alpha, Ovaleap, 300 IU (22 μg/0.5 ml)), diluted to a concentration of 300 ng/ml for 24 hours. In all experiments equal volume of PBS (Gibco) was included as control treatment. While the experiments were performed once, all conditions were performed in triplicate.

#### RNA isolation and real time polymerase chain reaction

Total RNA was extracted according to the TRI Reagent protocol by Sigma-Aldrich and total RNA concentrations were measured with the NanoDrop 2000 Spectrophotometer (Thermo Scientific). cDNA was synthesized with the iScript cDNA Synthesis Kit (Bio-Rad), for which a PTC-200 PCR machine was used. Real-time qPCR was performed according to the SensiFAST SYBR^®^ No-ROX Kit (Bioline). Gene expression levels were normalized for the combined value of glyceraldehyde 3-phosphatase dehydrogenase (*GAPDH*) and ribosomal protein lateral stalk subunit P0 (*RPLP0*) housekeeping genes. The used primer sequences are provided in **S1 Table**.

### THP-1 monocyte-like cell line

Since invasion of inflammatory cells and related vascular degradation are important histological features of AAA, and the FSHR has been reported to be expressed on monocytes/macrophages (12), we also performed qPCR on macrophages to detect a possible FSH effect. We used the THP-1 immortal monocyte-like cell line (ATCC^®^, catalog number TIB-202^TM^) for these experiments. Once the THP-1 cells had been grown in a suspension of RPMI 1640 (ThermoFischer)/10% FCS and there were a sufficient number of cells, these cells were added in a 6 well-plate. Once settled for 3 hours, the THP-1 cells were treated with 10 ng/ml of 12-O-tetradecanoylphorbol-I3-acetate (PMA) for 24 hours to differentiate into macrophages. Differentiated THP-1 cells were left untreated to be used as a reference well (control group) and compared with the activated differentiated macrophages. Differentiation of macrophages into a pro-inflammatory phenotype, characterized by the production of pro-inflammatory cytokines, was achieved by treating the PMA-differentiated macrophages with 15 ng/ml of lipopolysaccharide (LPS; Sigma E. coli E55:O5). Alternatively, PMA-differentiated macrophages were stimulated with FSH (100 ng/ml FSH). After 24 hours of FSH or LPS stimulation, the cells were lysed for RNA isolation to generate cDNA and to perform qPCR. *Beta-actin* (*ACTB*) and *RPLP0* were used as housekeeping genes. The primer sequences used are shown in **S1 Table**. While the experiment was performed once, all conditions were performed in triplicate.

### Human macrophages

The methods of differentiation of macrophages from human monocytes and stimulation of these cells has been described previously [16]. In short, buffy coats are acquired from healthy donors, from which monocytes are isolated and differentiated into macrophages (50 ng/ml human M-CSF (Miltenyi) in Isocove’s Moified Dulbecco’s Medium (IMDM) containing 10% heat-inactivated fetal bovine serum, 1% P/S and 1% L-glutamine solution (Gibco)). Subsequently, the human macrophages were left unstimulated (control group) or stimulated with LPS (10 ng/ml), interferon-γ (IFN-γ, 50 ng/ml, R&D), IL-4 (50 ng/ml, PeproTech), IL-10 (50 ng/ml, R&D) or dexamethasone (100 nM, Sigma) for 24 hours to assess if *FSHR* mRNA would be expressed under these conditions.

### Primers for FSHR detection

We developed a primer set to detect the human *FSHR*, based on the GenBank KR711783.1 (**S1 Table**). This primer set was used in testis cDNA to confirm that the primer set was able to detect the *FSHR*, which it did. In addition, we used three FSHR primer sets previously reported by Robinson *et al*. [12]. These authors determined that in ovarian tissue samples there is full length *FSHR* transcript, whereas in monocytes or monocyte-derived osteoclasts there is a shorter splice variant of *FSHR*, lacking exon 9. They developed different primer sets for which we use their nomenclature (primer sets 1, 2 and 5) [12]. The PCR product of primer set 1 spans exons 2–3 of which the PCR product is 120 base pairs (bp), which should be detected in similar size for both *FSHR* variants. Set 2 has one primer spanning the exon 8–10 border in the shorter variant and a primer in exon 10, generating a PCR product of 134 bp only for the short *FSHR* variant. Primer set 5 spans exons 8–10, which will generate a large product for the full length *FSHR* (320 bp) and a shorter product if exon 9 is missing (140 bp). The primer sets are given in **S1 Table**. cDNA samples at a concentration of 0.14–1 ng/μl isolated from testis, monocytes, HepG2 cells [17], macrophages, HUVECs, HUC SMCs, AAA tissue samples and AAA-derived SMC culture samples were used for real time qPCR with these FSHR primers. Amplification data and melting curves were analyzed with using Lightcycler 480 software (Roche) and LinRegPCR software. For electrophoresis of PCR products, a 2% agarose gel was prepared using agarose (Invitrogen, 16500–500) dissolved in 1x Tris-Acetate EDTA (TAE) buffer (40 mM Tris-acetate, 1mM EDTA) mixed with 1:20 000 diluted ethidium bromide (Sigma) to visualize the DNA. After solidification, amplicons were mixed with 10x FastDigest Green buffer and 8μl was loaded in each lane, flanked with 5μl Generuler DNA ladder mix (Thermo). Electrophoresis was performed using a horizontal system (Bio-Rad) for 45 minutes at 100V. Gels were imaged with a GenoSmart imaging system (VWR).

### AAA dataset and AAA samples

In AAA it is more relevant to assess if there is *FSHR* gene expression in human AAA tissue and control aortic tissue. To that end, we consulted the available human AAA datasets (NCBI GEO database GSE 98278 and GSE57691) [18,19]. The differential expression of the *FSHR* gene was assessed between elective AAA tissue and control aortic tissue, and *FSHR* gene expression was compared to all measured transcripts. Since expression levels were measured as an intensity, absolute values of expression could not be given. In addition, differential *FSHR* gene expression was assessed between elective and ruptured AAA, and large versus intermediate AAA. Since gene expression was measured multiple times by different probes of the microarray, the minimum *P* value of the gene was selected for reporting.

Aortic aneurysm biopsies were obtained during open AAA repair at the Amsterdam University Medical Centers, and Dijklander Hospital, Hoorn, The Netherlands, in accordance with the Declaration of Helsinki and institutional guidelines. Written informed consent to participate in the study was obtained from all patients. Experiments and experimental protocols were performed in accordance with institutional guidelines and approved by the Medical Ethics Committee of the VU Medical Center.

qPCR was performed on cDNA of human AAA tissue samples (from 4 elderly women) and human AAA-derived cultured SMCs (from 4 elderly women) to assess *FSHR* gene expression. AAA tissue was snap-frozen in liquid nitrogen and stored at -80°C until RNA isolation with TRIzol^TM^ Reagent (Thermo Fischer Scientific). cDNA was synthesized using iScript cDNA Synthesis Kit (Bio-Rad Laboratories). SMC were isolated from AAA biopsies as described in Bogunovic *et al*. [20] and cultured in 231 medium (Thermo Fisher Scientific, Walthem, MA, USA), supplemented with Smooth Muscle Growth Supplement (Thermo Fisher Scientific), and 100 units/ml penicillin and 100 μg/ml streptomycin (Thermo Fischer Scientific) in a humidified incubator 37°C, 5% CO2. The cells were lysed with lysis buffer (Quick-RNA miniprep Kit, Zymo Research, Irvine, CA) and RNA purification was performed following the protocol of Quick-RNA miniprep Kit (Zymo Research, Irvine, CA). cDNA synthesis was performed according to the instructions of the SuperScript VILO cDNA synthesis kit (ThermoFischer Scientific), using the T100 ThermalCycler (Bio-Rad Laboratories, Veenendaal, The Netherlands).

Since *FSHR* was detected on gel electrophoresis in occasional AAA-derived SMC culture samples, FSH stimulation experiments were conducted with four female AAA-derived SMCs in duplo for each condition. FSH was added (300 ng/ml FSH for 24 hours) once the cells became confluent. Subsequently, gene expression for *FSHR* and indicated markers was measured by qPCR and acquired using CFX384 Touch Real Time PCR detection system (Bio-Rad Laboratories). *RPLP0* was used as housekeeping gene.

In addition, we stimulated *FSHR* positive and *FSHR* negative AAA SMCS with FSH for 15/60/120 minutes to assess differences in phosphorylated AKT (p-AKT) and total AKT (t-AKT) with Western blot. For feasibility reasons, we used the two AAA SMCs in which we detected the *FSHR* on gel and the AAA SMCs in which the *FSHR* was absent. Human aortic SMCs were lysed with ice cold RIPA buffer supplemented with protease (cOmplete, Mini, EDTA free; Merck) and phosphatase (PhosSTOP; Sigma-Aldrich) inhibitors. The supernatant was collected after 10 minutes centrifugation at 12 000rpm for aortic SMCs. Protein measurements were performed with the Bio-Rad DC assay according to manufacturer’s instructions. Equal amounts of volume were loaded on a 10% SDS polyacrylamide gel, and upon gel electrophoresis protein was transferred onto a nitrocellulose membrane with the Bio-Rad Transblot Turbo transfer system. After one-hour’s incubation with blocking buffer (2% milk powder in TBST) at RT, the membranes were incubated overnight at 4°C with a 1:1000 dilution of primary antibody against p-AKT, or t-AKT (Cell Signaling) after stripping of the blot. The blots were also stained for 1:1000 β-actin (Cell Signaling) as a loading control. For band detection, membranes were incubated with the corresponding secondary HRP labeled IgG followed by a 5 minute incubation with SuperSignal™ West Pico PLUS chemiluminescent substrate (ThermoFisher 34580) and visualized with ImageQuant 800 Western blot imaging system (Amersham).

### Human monocytes

#### Monocyte isolation and FSH stimulation

To explore if the THP-1 cells may have lost some of the characteristics of ‘healthy and fresh’ monocytes, and if circulating monocytes could be stimulated under a functional FSHR at protein level, we continued our research with monocytes isolated from the buffy coats of healthy anonymous blood donors. Peripheral blood mononuclear cells were isolated from 4 donors (2 males, 2 females) from buffy coats (Sanquin Blood Bank, Amsterdam, The Netherlands) through density centrifugation using Lymphoprep (Axis-Shield). All donors provided written informed consent in accordance with the protocol of the local institutional review board, the Medical Ethics Committee of Sanquin blood supply (Amsterdam, The Netherlands, contract number: NVT0179.00) in accordance with the Declaration of Helsinki. Monocytes were then purified using human CD14 magnetic beads and MACS cell separation columns (Miltenyi Biotec). Monocytes were cultured in suspension culture plates at a density of 1*10^6^ cells/mL in Iscove’s modified Dulbecco’s Medium (IMDM, Sigma-Aldrich, Zwijndrecht, The Netherlands) supplemented with 2mM l-glutamine, penicillin (100 U/mL), streptomycin (100 ug/mL) and 10% FCS (all Gibco). Monocytes were stimulated overnight with FSH 500 mIU/mL (36.7 ng/ml) or left unstimulated.

#### Ox-LDL uptake

In a previous study the higher lipid content was shown to coincide with increased cell surface expression of CD36 on classical monocytes [21]. Thus, since CD36 is involved in oxidized low-density lipoprotein (ox-LDL) uptake, we used the CD14 beads isolated monocytes (Miltenyi Biotec, Bergish Gladbach, Germany) to assess differences in ox-LDL uptake and the upregulation of activation markers with and without FSH stimulation. Ox-LDL uptake was measured by use of DiI labeled ox-LDL (Thermofisher). Media of FSH stimulated or unstimulated monocytes were replaced by fresh medium and 5ug/ml DiI labeled ox-LDL was added for 3 hours allowing uptake [22]. Hereafter, the cells were washed with PBS and lifted with TripLE (Thermofisher) for flow cytometry. Ox-LDL uptake was measured by flow cytometry by measuring fluorescence in the PE Channel (Beckman Coulter CytoFLEX) and analyzed by use of FlowJo software (version 10.7.0.). Debris and doublets were excluded using forward scatter (FSC) and side scatter (SSC). Ox-LDL uptake was presented as a percentage of ox-LDL positive cells, and median fluorescence intensity (MFI) was used to measure the amount of ox-LDL uptake per cell.

#### Flow cytometry

We assessed if FSH stimulation impacted the expression of surface markers on human monocytes by flow cytometry. The methods of flow cytometry in monocytes have been described before (21, 22) and were adjusted to our FSH stimulation experiments. FSH stimulated or unstimulated monocytes were washed with PBS and lifted with TripLE for flow cytometry. FC receptors were blocked with intravenous immunoglobulin (IVIG, Sanquin) for 15 minutes at room temperature. Monocytes were stained for 20 minutes at room temperature with fluorescent labeled antibodies against CD36 (PE, BD Bioscience), CD64 (APC, Biolegend), CD14 (PE-Cy7, BD Bioscience), CD16 (APC-Cy7, BD Bioscience) and HLA-DR (MHC-II) (PerCP-Cy5.5, BD Bioscience). Cells were washed and resuspended in FACS buffer (PBS with 0.5% BSA and 2.5 mM EDTA) and measured on a Beckman Coulter CytoFLEX and analyzed with FlowJo software (version 10.7.0.). Debris and doublets were excluded based on FSC/SSC and CD14+/CD16+ cells were considered as monocytes. The expression of the surface markers was presented as MFI, representing median marker expression per cell.

### Statistical analyses

Differences between two groups in gene expression analyzed by PCR or differences in markers assessed by flow cytometry analysis were assessed with the independent t-test or Mann-Whitney U test, as appropriate. For differences between ≥ 2 groups, the one-way ANOVA test with Tukey post-hoc-test or Kruskal Wallis test was performed for normally or skewed distributed data, respectively. A *P* value of (α = 0.05/7 = 0.007 for EC, α = 0.05/6 = 0.008 for SMC, α = 0.05/7 = 0.007 for THP-1 cells, α = 0.05/5 = 0.01 for AAA-SMCs) was considered as statistically significant to account for multiple testing. Statistical analyses were conducted in IBM SPSS Statistics 26.

## Results

### Effect of FSH on endothelial cells and smooth muscle cells

To assess if FSH can induce a gene expression pattern representing EC or SMC activation, both cell types were stimulated *in vitro*. FSH stimulation induced similar *IL1B*, *IL6*, *CXCL8* or *CCL2* gene expression in HUVECs and HUCs when compared to control (**S1A and S1B Fig**). Regarding cell-specific markers, *ICAM1* and *VEGF* gene expression in HUVECs were similar, whereas a downregulation of the contractile gene *ACTA2* in HUCs (*P* = 0.007) was observed. However, while *FSHR* gene expression was detected in testis tissue—used as positive control—it was undetectable in HUVEC and HUC samples (PCR cycles >32; **S1C Fig**).

### Effect of FSH on THP-1 monocyte-derived macrophages

While LPS significantly induced all tested genes, FSH did not change gene expression of any of the selected genes significantly; *IL1B*, *TNF*, *CXCL12*, *NOS2*, *TGFB1*, *MMP9*, or *CTSS* (**S2 Fig**). There was only a downregulation of *TNF* (*P* = 0.039), *NOS2* (*P* = 0.044) and *MMP9* (*P* = 0.027) gene expression by FSH, which was not significant after correction for multiple testing (*P* >0.007). Again, *FSHR* gene expression was below detection level (PCR cycle >32; **S3 Fig**).

### FSHR expression in HUVECs, HUCs, monocytes, macrophages and HepG2 cells

**Fig 1:**
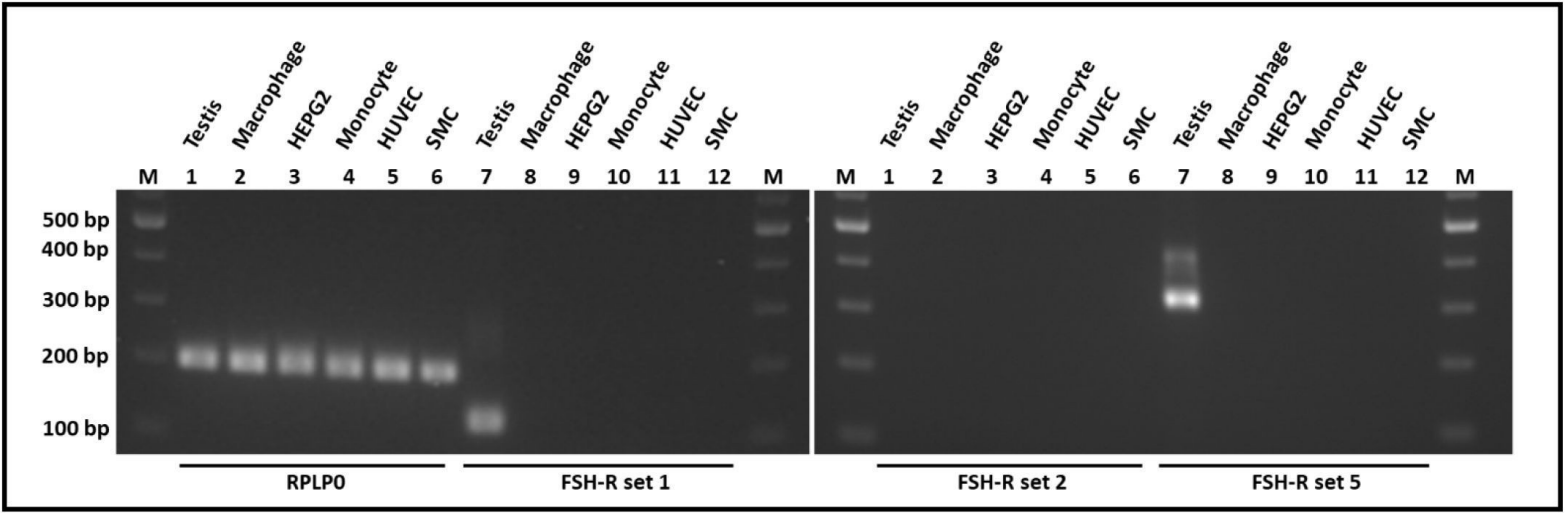
*FSHR* gene expression in extragonadal samples. Testis and five extragonadal tissues were tested for follicle stimulating hormone receptor (FSHR) expression of the full length or short variant of *FSHR* by PCR with three different primer sets, as described by Robinson *et al*. (12). Primer set 1 detects full length *FSHR*, set 2 detects a truncated variant and set 5 can detect both. Housekeeping gene RPLP0 was adequately expressed in all cells. Testis tissue gave a PCR product of 120 base pairs (bp) with primer set 1 and 320 bp with primer set 5, indicating it only produces the full length *FSHR*. None of the extragonadal cells (human monocyte-derived macrophages, cell line HepG2 liver cells, primary human monocytes, human umbilical vein endothelial cells (HUVECs) or human umbilical cord artery smooth muscle cells (HUC SMCs)) produced a detectable *FSHR* PCR product.

The *FSHR* is expressed in testis and can be detected with primer sets 1 and 5, generating a single PCR product reflecting the full length *FSHR* (**Fig 1**). Since only Sertoli cells express the *FSHR* in testis, the expression was low as determined by detection at PCR cycles around 29–30 for primer set 1 and 31–32 for primer set 5. However, no *FSHR* could be detected as full length or shorter variant in human monocyte-derived macrophages, HepG2 liver cells, primary monocytes, HUVECs or HUC SMCs (**Figs 1 and S4**).

To further explore if the FSHR may be expressed more prominently upon activation, we assessed human monocyte-derived macrophages incubated with LPS, IFN-γ, IL-4, IL-10 or dexamethasone. Unstimulated macrophages were used as control. No *FSHR* gene expression was detected under any of these conditions (**S5 Fig**).

### FSHR expression and FSH stimulation in AAA

**Fig 2:**
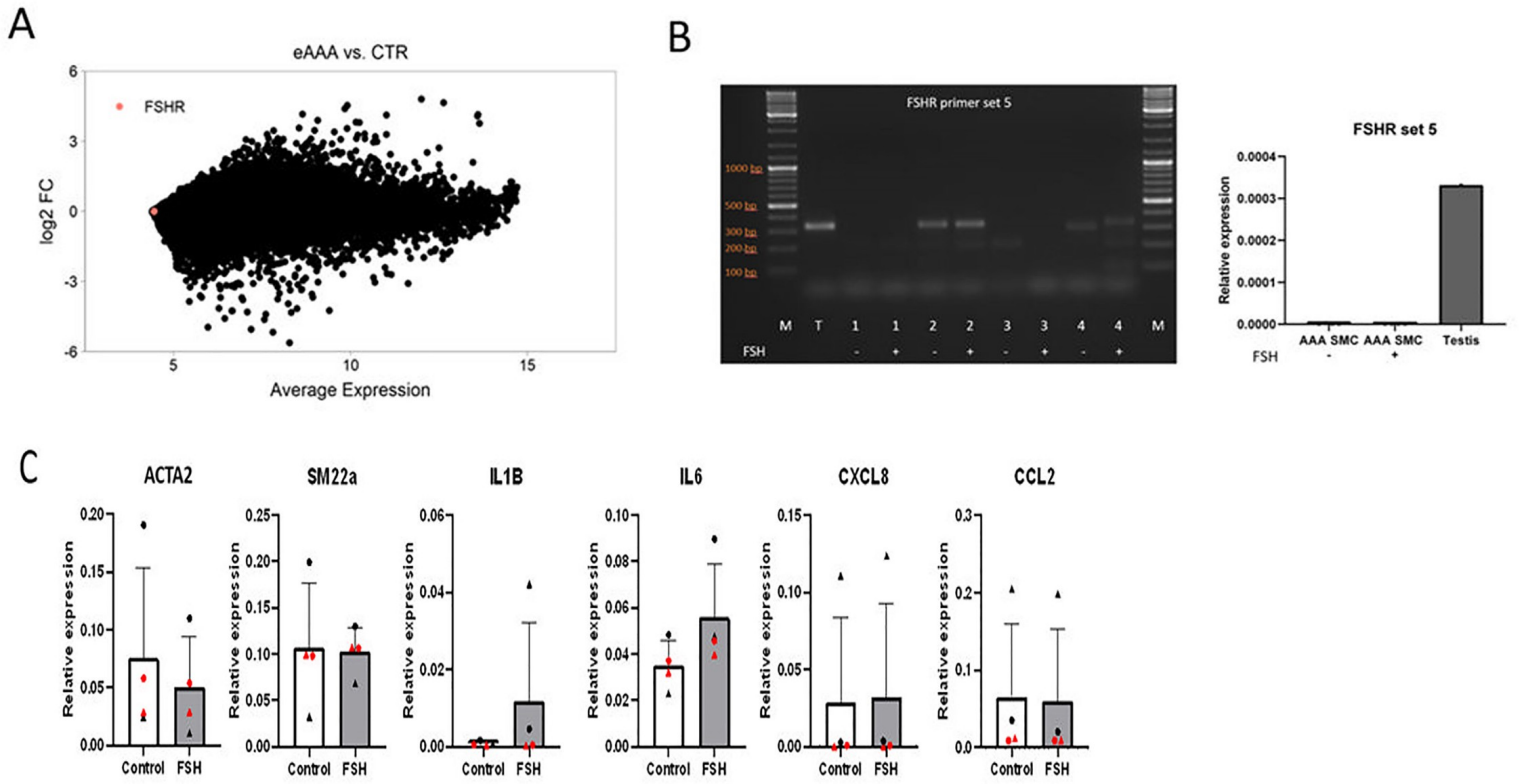
*FSHR* gene expression in AAA samples. The microarray dot-plot derived from public AAA datasets (NCBI GEO database GSE 98278 and GSE57691) shows that the *FSHR* expression is not differentially expressed in AAA tissue from patients undergoing elective open repair (eAAA) versus control aortic tissue (CTR) from organ donors, and that the *FSHR* expression is low compared to the average gene expression of all available transcripts (A). AAA-derived SMCs of 4 female patients were stimulated with and without FSH. qPCR with primer set 5 revealed that donors 2 and 4 have bands that are at similar height as the testis sample (T), suggesting these two SMC lines may have FSHR expression of the full length receptor (gel), while the signal appeared only after PCR cycle 32 rendering the quantification as too low to quantify (graph) (B). Each donor represents either a black circle, black triangle, red circle or red triangle. Gene expression did not change significantly upon FSH stimulation for various markers (4 donors; average of duplo per condition), especially not in the SMC lines that showed FSHR expression, as indicated in red (C). ACTA2 = alpha SMC actin; SM22a = SMC22alpha; IL1B = Interleukin-1beta; IL6 = Interleukin-6; CXCL8 = Interleukin-8; CCL2 = monocyte chemoattractant protein-1.

The AAA dataset of Gäbel *et al*. [18] showed that *FSHR* gene expression was not differentially expressed in AAA tissue from patients undergoing elective repair than in control aortic tissue from organ donors, and that there was very low *FSHR* gene expression in both tissues on comparison with the average gene expression (**Fig 2A**). In addition, there was no difference in *FSHR* gene expression in ruptured AAA tissue versus elective AAA tissue (adjusted *P*_*min*_ = 0.72), or in large AAA (>70mm) versus intermediate AAA (≤55mm) (adjusted *P*_*min*_ = 0.91).

As these datasets analyzed the data of relatively few women, the possibility exists that *FSHR* gene expression is different in women. Therefore, in our further experiments we focused on female donors. While *FSHR* gene expression could not be detected in the female AAA tissue samples (primers set 1 and 5; PCR cycles >32; **S6 Fig**), it was only detected by gel electrophoresis in two out of 4 female AAA-derived SMC cultures by putting the PCR product on gel (**Figs 2B and S7**). However, FSH stimulation experiments in these AAA-SMCs did not affect gene expression levels of *ACTA2* (*P* = 0.564), *SM22a* (*P =* 0.913) *IL1B (P = 0*,*100)*, *IL6 (P = 0*.*153)*, *CXCL8 (P = 0*.*773) or CCL2 (P = 0*.*564)* (**Fig 2C**), since the cells with FSHR expression on gel showed the least variability upon FSH. In addition, assessment by Western blot showed that FSH stimulation (various time duration) had no effect on phosphorylated AKT in FSHR+ and FSHR- AAA-SMCS (**S8 Fig**).

### Effect of FSH on primary human monocytes

**Fig 3:**
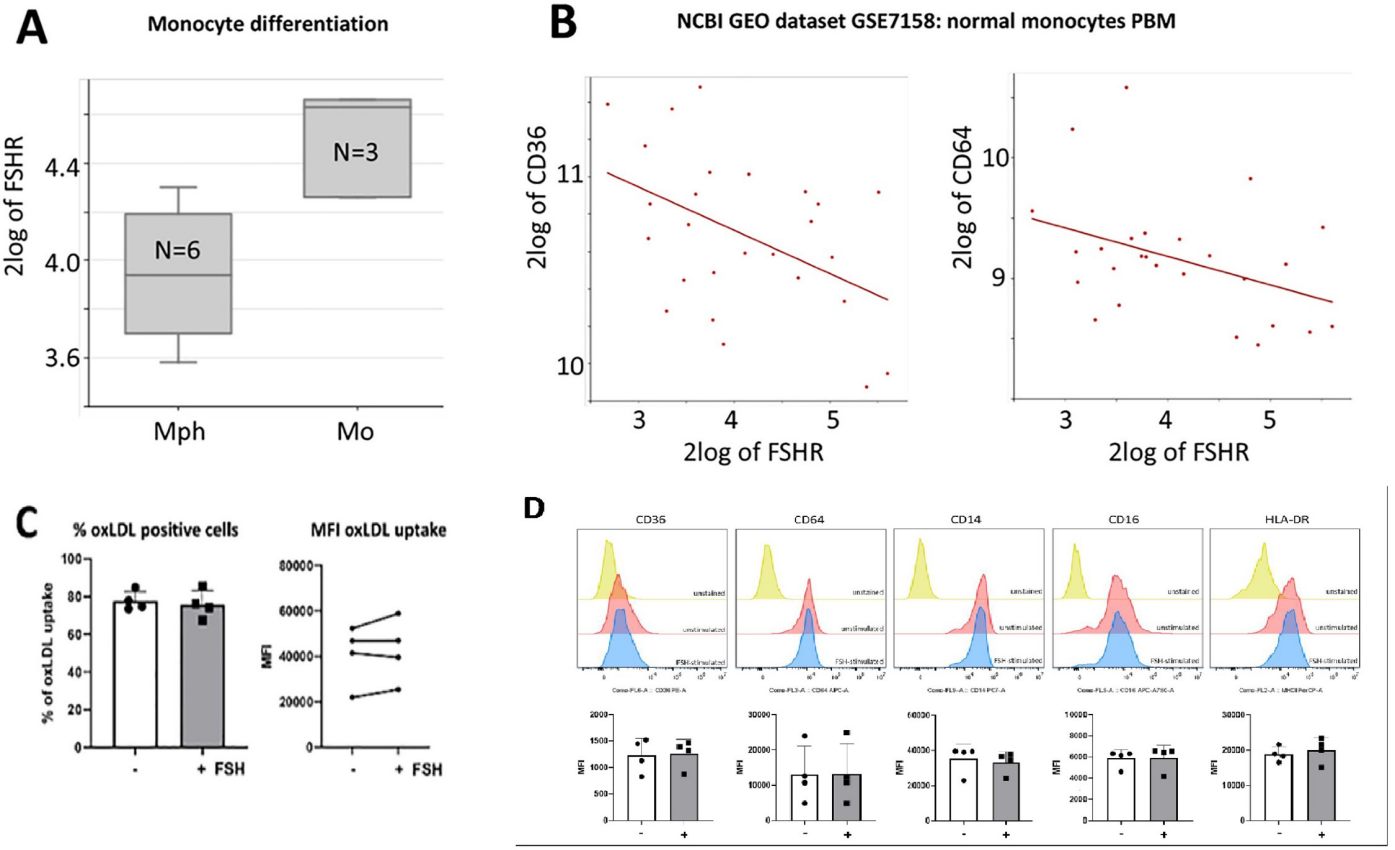
FSH stimulation of human monocytes. R2 analysis of macrophages (Mph) and monocytes (Mo) shows enhanced follicle stimulating hormone receptor (FSHR) expression in monocytes compared to macrophages (A). A negative association is observed between *FSHR* and *CD36* expression (*P* = 0.018) as well as *CD64* expression (*P* = 0.047) in monocytes derived from 26 healthy donors (B). Flow cytometry data of monocytes from 4 different healthy donors (all conditions in triplicate) show no difference in ox-LDL positive cells, nor in in the amount of ox-LDL uptake (expressed as median fluorescent intensity; MFI) after FSH stimulation (C). Expression of cell surface markers on healthy monocytes after FSH stimulation (D). Yellow represents the unstained monocytes (negative control), red the unstimulated monocytes and blue the FSH stimulated monocytes both incubated with the indicated antibodies. Graphs of cell surface marker expression is the average of 4 blood donors (D). White bars represent the unstimulated group and gray bars the FSH stimulated group (C and D).

If local cells within the aorta, such as ECs, SMCs and macrophages do not show prominent *FSHR* gene expression and FSH response, then perhaps we should focus on the monocytes just before they may enter the vessel wall. Therefore, we aimed to explore if circulating monocytes could be activated before becoming macrophages. We used the R2 platform which is developed at the Amsterdam UMC and offers the possibility to explore public gene expression datasets (R2: Genomics Analysis and Visualization Platform (http://r2.amc.nl)). We found in the dataset of Ismail *et al*. that monocytes express more *FSHR* mRNA than monocyte-derived macrophages [23] (**Fig 3A**). Moreover, in a dataset of primary human monocytes from 26 healthy Chinese women aged 20–45 years with either high or low peak bone mass as indicator for potential osteoporosis (NCBI Gene Expression Omnibus GEO ID; GSE7158), we observed that there was an inverse correlation between *FSHR* and *CD36* or *CD64* mRNA expression (*P* = 0.018 and *P* = 0.047, respectively) (**Fig 3B**). We could not find an association between *FSHR* and *ABCA1*, *HMGCR*, *CD9*, *CD11a*, *CD11b*, *CD11c*, *CD14*, *CD33*, *CD43*, *CD45*, *CD61*, *CD93*, *HLA-DRA or–BI*, *CCR2*, *CCR5* or *CXCR1* expression (examples are given in **S9 Fig**), which we searched for as they are typical monocyte markers or relevant in AAA.

CD36 is recognized to be involved in the uptake of ox-LDL by monocytes and macrophages [24], leading to the formation of foam cells, which plays its part in atherosclerosis [25]. Therefore, we used CD14 beads-isolated monocytes from four individual healthy blood donors to assess differences in ox-LDL uptake and cell surface protein expression of various markers upon FSH stimulation. After flow cytometry no differences could be detected in the percentage of ox-LDL positive cells or in median ox-LDL uptake per cell between FSH stimulated and unstimulated human monocytes (**Fig 3C**). Furthermore, cell surface expression of the protein markers CD36, CD64, CD14, CD16 or HLA-DR (MHC-II) between FSH stimulated and unstimulated monocytes was not different (**Fig 3D**), revealing no shift in marker expression by FSH.

## Discussion

While the current study was interested in identifying any potential *FSHR* gene expression in AAA tissue and cell types, and the potential effect of FSH stimulation as a possible explanation of enhanced AAA progression in postmenopausal women, it has become clear that there is no significant *FSHR* gene expression or FSH effect to justify further exploration of this hypothesis. We detected a downregulation of *ACTA2* in HUC SMCs after FSH stimulation. However, this was not seen in AAA-SMCs. Furthermore, FSH stimulation did not significantly influence gene expression in HUVECs or THP-1 macrophages, or have an effect on ox-LDL uptake by primary human monocytes or expression of any of the cell surface markers investigated. *FSHR* transcripts could not be detected in HUVECs, HUC SMCs, PMA-differentiated THP-1 macrophages, primary human monocytes, monocyte-derived macrophages and AAA tissue samples. Moreover, macrophages incubated with various stimuli also did not lead to detection of the *FSHR*. We only observed a band on gel after PCR, corresponding to full length *FSHR* expression, in two out of four female AAA-derived SMC cultures. Yet, these cells did not respond to an FSH stimulus. Moreover, we did not detect the *FSHR* in human HepG2 cells—another type of extragonadal cell reported to have the FSHR [17]—while it was detected in testis tissue. Therefore, we explain the lack of convincing effect of FSH stimulation on the cell types we used in our *in vitro* studies by the absence or insufficient *FSHR* gene expression to generate a response.

We could not replicate the findings of Stilley *et al*. who showed that FSH stimulation of HUVECs resulted in angiogenesis, reflected by upregulation of *VEGF* [11]. As our umbilical cord material was received anonymously, we were unable to establish if our cells were of male or female infant donor origin; this is a point of consideration for a future study. However, we corroborated the finding of Stelmaszewska *et al*. [26] as we did not detect *FSHR* mRNA in our HUVECs, which encodes for the FSHR at the protein level. This may partly explain why FSH could not exert its effect. However, we detected reduced *ACTA2* gene expression in HUC SMCs although no *FSHR* mRNA was detected in these cells. Interestingly, prior work has reported a discrepancy between mRNA and protein detection of the *FSHR* [26]. The authors showed that while detection of *FSHR* transcripts could not be established, the FSHR was identified with anti-FSHR antibody FSH323 in HUVECs, and in ovarian tissue as positive control [26]. This finding may imply that the FSHR at the protein level is very stable, and low mRNA expression is sufficient to maintain receptor presence. In this case, the researcher is highly dependent on the quality of the anti-FSHR antibody. However, a critical review recently reported that current data on extragonadal FSHR localization are often based on insufficiently verified antibodies used in immunohistochemistry, thus challenging the interpretation of these data. Suggestions on how to properly validate anti-FSHR antibodies are given by the authors [27].

Alternatively, *FSHR* expression may be difficult to capture as *FSHR* transcripts have been reported to have low expression in HUVECs, osteoclasts and monocytes as compared to ovary [12,13]. As in our testis samples *FSHR* expression was already quite low because it is only expressed in Sertoli cells, *FSHR* gene expression is possibly more difficult to assess in extragonadal cells, even though we used one single cell type per condition. Additionally, it has been shown that the *FSHR* has a different splice variant lacking exon 9 in certain extragonadal cells [11,12]. Therefore, we used the primer sets designed by Robinson *et al*. [12] to distinguish the different *FSHR* transcripts, and could detect only full length *FSHR* mRNA transcript in testis and in two out of four cultured female AAA-SMCs, but not in any of the other explored extragonadal cells.

As the lack of effect of FSH stimulation and *FSHR* gene expression did not give cause to repeat experiments on HUVECs, HUCs and THP-1 cells, the experiments were performed only once in triplicate, and this is a limitation in our study. Another limitation of the study is that we did not perform cAMP analysis for the PKA pathway. Instead, to further pursue the question if FSH could have a direct effect in AAA, we aimed to detect *FSHR* gene expression in AAA. However, *FSHR* gene expression was not seen in AAA tissue. While two out of four female AAA-derived SMC lines did reveal FSHR expression at a very low level, FSH stimulation of AAA-SMCs did not result in differences in levels of gene expression of various SMC or activation markers. However, the data should be interpreted with caution as the sample size was small. Repetition of the experiment with a larger sample size would provide a more robust conclusion.

The meaning of the negative correlation between the expression of *FSHR* and *CD36* or *CD64* in peripheral blood monocytes was explored by first assessing if we could find a potential impact of FSH on ox-LDL uptake or upregulation of CD64, reflecting a pro-inflammatory phenotype of the monocyte [28,29]. CD36 on monocytes/macrophages has been identified as an important factor in atherosclerosis through binding and promoting endocytosis of ox-LDL [30]. As such, it is implied in the formation of foam cells. We were interested to see if FSH stimulation through the FSHR could enhance the induction of a pro-inflammatory monocyte, reflected by an increase in ox-LDL uptake and/or increase in CD36 or CD64 expression. However, we did not find that FSH influenced ox-LDL uptake, suggesting that FSH does not impact foam cell formation directly, which affects cardiovascular disease. Moreover, the cell surface protein expression of CD36 and CD64 were not altered upon FSH stimulation, similarly to the expression of the other investigated cell surface markers, revealing no sign of activation by FSH of these freshly isolated human monocytes.

Although *FSHR* gene expression has been described as functional in other extragonadal cells [31–33], our findings show that we currently have no indication that FSH stimulation on the FSHR has a direct effect on the enhanced AAA development in women during the menopausal transition. We recently published a review on this topic identifying the potential direct and indirect ways that FSH could enhance AAA [10]. Our current findings lead us to consider that it may be more plausible that the indirect effects of FSH could enhance AAA progression rather than its direct effects on the vascular cell types assessed in this study. FSH may affect AAA indirectly through FSH-induced osteoporosis that is associated with vascular calcification, increased cholesterol levels due to reduced liver uptake of LDL that can promote aortic atherosclerosis and inflammation, and FSH-induced adipogenesis leading to microvascular dysfunction and/or modulation of periaortic adipose tissue. The concept that FSH may potentially impact extragonadal cells and that this may result in postmenopausal disease in women is highly interesting. However, further research is required to demonstrate *FSHR* expression and/or localization in non-vascular cell types, and the impact of FSH stimulation on these *FSHR* positive extragonadal cells in the context of AAA.

## Conclusion

The current study was an extensive attempt to detect the *FSHR* in extragonadal cells of the vasculature and inflammatory cells known to be present in AAA tissue, based on the identification of FSHR expression in endothelial cells and monocytes/macrophages as described by others [11,12]. However, we could not reproduce these data, nor did we find significant *FSHR* expression in AAA-derived SMCs or in AAA tissue. Moreover, we found no significant changes in gene expression upon FSH stimulation. Thus, we conclude that we currently have no clues for further research into the hypothesis that prolonged high FSH levels in postmenopausal women can directly affect AAA tissue or AAA-related cell types via the FSHR.

## Supporting information

S1 Fig Gene expression in HUVECs and HUCs upon FSH stimulation. Gene expression in human umbilical vein endothelial cells (HUVECs) (A) and human umbilical cord artery smooth muscle cells (HUC SMCs) (B) upon FSH stimulation. A downregulation of *ACTA2* in HUC SMCs was observed after FSH incubation (*P* = 0.007). IL = interleukin; CXCL8 = C-X-C motif chemokine ligand 8; CCL2 = C-C motif chemokine ligand 2; ICAM1 = intercellular adhesion molecule 1; VEGF = vascular endothelial growth factor; ACTA2 = smooth muscle specific alpha actin; FSHR = follicle stimulating hormone receptor.
(TIF)

S2 Fig Gene expression in THP-1 PMA-induced macrophages upon LPS or FSH stimulation. The control group were PMA-differentiated macrophages. LPS potently induced expression of all genes measured and a non-significant downregulation of *TNF* (*P* = 0.039), *NOS2* (*P* = 0.044) and *MMP9* (*P* = 0.027) was observed after FSH incubation. IL = interleukin; TNF = tumor necrosis factor; CXCL12 = C-X-C motif chemokine ligand 12; NOS2 = nitric oxide synthase 2; TGFB1 = transforming growth factor β 1; MMP9 = matrix metalloproteinase 9; CTSS = cathepsin S. PMA = phorbol 12-myristate 13-acetate; LPS = lipopolysaccharide; FSH = follicle stimulating hormone.
(TIF)

S3 Fig qPCR output of THP-1 monocyte-derived macrophages upon PMA, after stimulation with LPS, FSH or control. β-actin and RPLP0 were used as housekeeping genes. The data reveal that Cq cycles for FSHR were beyond our threshold of 32, while for example cytokines IL-1β and TNFα are abundantly expressed with low Cq values.
(TIF)

S4 Fig qPCR output of various extragonadal cells as shown in Fig 1 of the manuscript. RPLP0 was used as housekeeping gene. The nomenclature of Robinson et al. was used for FSHR_set1, FSHR_set2 and FSHR_set 5. FSHR primer set 2 should only recognize the shorter FSHR variant which has been observed previously in extragonadal cells. However, here we do not see any relevant FSHR expression with any of the FSHR primer sets, except the normal FSHR variant in testis with FSHR primer set 1 (which only recognizes the normal FSHR) and set 5 (which should recognize the normal and short FSHR variant).
(TIF)

S5 Fig qPCR output of two donors (D23 and D24) monocyte-derived macrophages stimulated with various stimuli for *FSHR* gene expression. The various stimuli were control (Mo), LPS (mLPS), interferon- γ (INFY), IL-4 (IL4), IL-10 (IL10) and dexamethasone (DEX). Testis was used as positive control. RPLP0 was used as housekeeping gene. The data show that there was no amplification of FSHR transcripts in the macrophages, irrespective of being stimulated by various stimuli. These were samples from a previously published study: DOI: 10.3389/fimmu.2019.02887.
(TIF)

S6 Fig qPCR output of AAA tissue samples (aorta) and AAA-derived SMCs for *FSHR* gene expression. FSHR_1 stands for primer set 1 and FSHR_5 for primer set 5 (nomenclature of Robinson et al.). RPLP0 was used as housekeeping gene. The data reveal that no *FSHR* transcripts were detected in AAA tissue and that in AAA-SMCs the Cq was beyond our Cq threshold (A). The PCR products on gel of RPLP0 as housekeeping gene (1) and *FSHR* (2; primer set 1) of AAA samples and AAA-derived SMCs. M stands for marker, T for testis (positive control), Ao for the AAA samples and SMC for the AAA-derived SMCs. The data show that the housekeeping gene is detected in all samples, and that the FSHR is only detected in testis and in two AAA-SMCs (B).
(TIF)

S7 Fig qPCR output of four AAA-SMCs cell lines from postmenopausal women stimulated with (+) FSH and without (-) FSH for *FSHR* gene expression. Testis RNA was used as positive control. RPLP0 was used as housekeeping gene. FSHR set 1 and FSHR set 5 correspond to the nomenclature of Robinson et al. and show high Cq values or lack of amplification for FSHR transcripts. *ACTA2* was used as a typical smooth muscle cell marker with relatively high expression.
(TIF)

S8 Fig Western blot of phosphorylated and total AKT after FSH stimulation. Western blot of phosphorylated AKT (p-AKT, Ser473) and total AKT (t-AKT) after FSH stimulation (FSH) versus control (C) for 15-60-120 minutes in AAA-SMC with (+) FSHR and AAA-SMC without (-) FSHR expression from postmenopausal female donors. Beta-actin was used as control. No regulation is observed in AKT phosphorylation by FSH in SMCs with or without FSHR expression.
(TIF)

S9 Fig Association between FSHR and various typical monocyte markers. Exploration using the R2 platform (http://r2.amc.nl) for an association between FSHR and various typical monocyte markers in circulating monocytes from 26 females (age 20–45) with either high or low bone mass (GSE7158). First, there is variable FSHR expression on monocytes of these 26 females. Secondly, CD36 expression in these monocytes was significantly inversely correlated with FSHR expression. All other markers are examples that these markers did not associate with FSHR expression in monocytes. ABCA1 = ATP-binding cassette transporter-1; CCR2 = C-C motif chemokine receptor type 2 or MCP1 receptor; CCR5 = C-C motif chemokine receptor type 5 or RANTES receptor; HMGCR = 3-hydroxy-3-methylglutaryl coenzyme A (HMG-CoA) reductase.
(TIF)

S1 Table Human primer sequences used for real-time quantitative polymerase chain reaction. (DOCX)

S1 Raw images (TIF)

S2 Raw images (TIF)

S3 Raw images (TIF)
