# Supplementary figures and images for: Exploring the expression and potential function of follicle stimulating hormone receptor in extragonadal cells related to abdominal aortic aneurysm
Source: PLoS One. 2023 May 25;18(5):e0285607. doi: 10.1371/journal.pone.0285607 (PMC10212101; doi:10.1371/journal.pone.0285607)

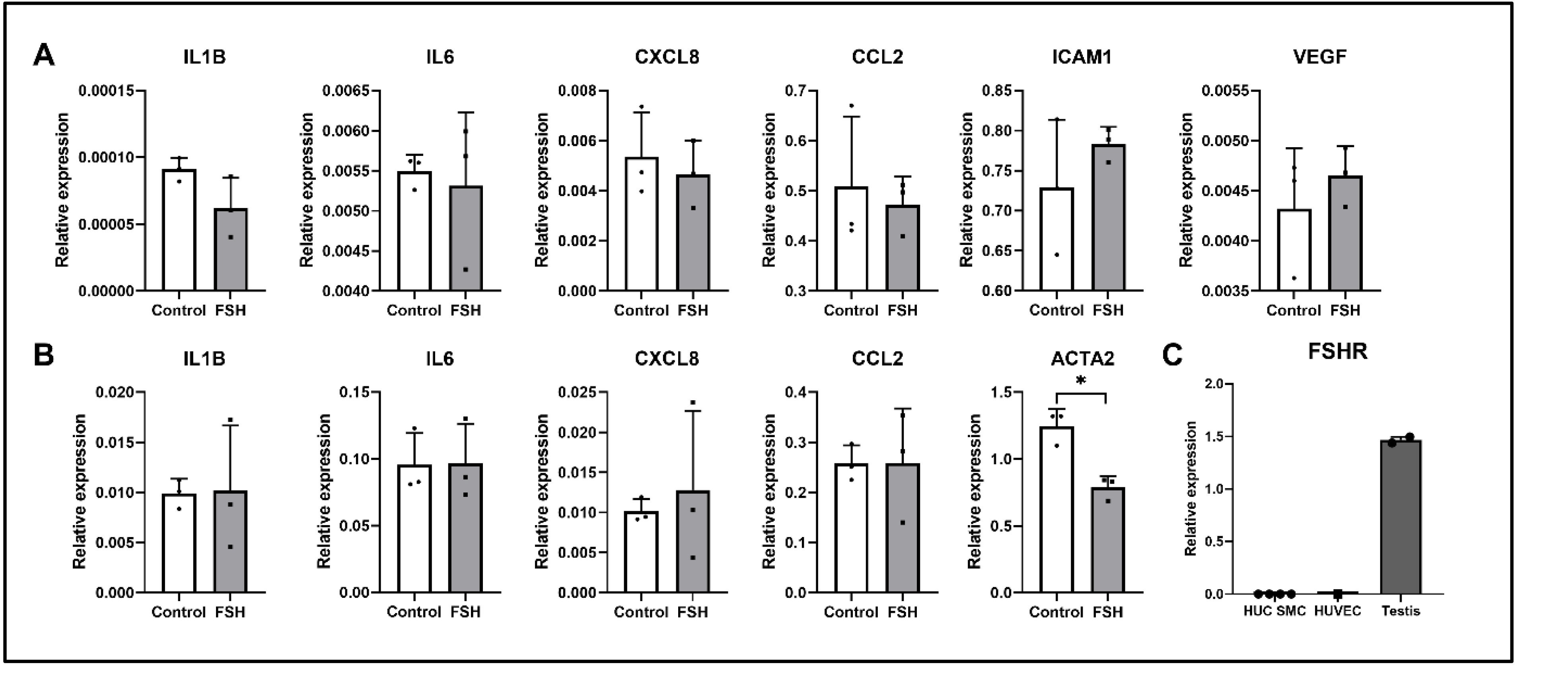

Supplement: S1 Fig — Gene expression in human umbilical vein endothelial cells (HUVECs) (A) and human umbilical cord artery smooth muscle cells (HUC SMCs) (B) upon FSH stimulation. A downregulation of ACTA2 in HUC SMCs was observed after FSH incubation (P = 0.007). IL = interleukin; CXCL8 = C-X-C motif chemokine ligand 8; CCL2 = C-C motif chemokine ligand 2; ICAM1 = intercellular adhesion molecule 1; VEGF = vascular endothelial growth factor; ACTA2 = smooth muscle specific alpha actin; FSHR = follicle stimulating hormone receptor. (TIF) [file pone.0285607.s001.tif]

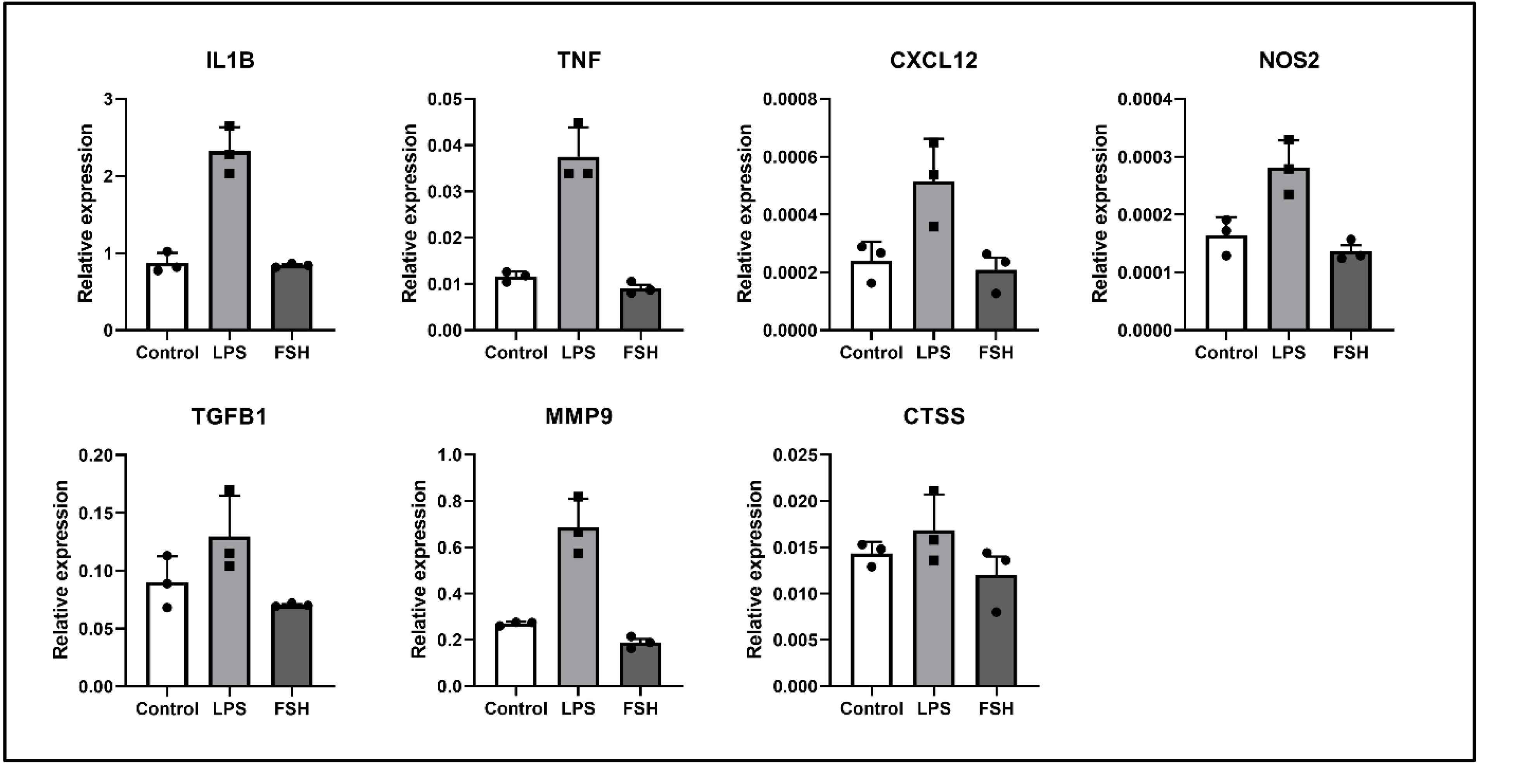

Supplement: S2 Fig — The control group were PMA-differentiated macrophages. LPS potently induced expression of all genes measured and a non-significant downregulation of TNF (P = 0.039), NOS2 (P = 0.044) and MMP9 (P = 0.027) was observed after FSH incubation. IL = interleukin; TNF = tumor necrosis factor; CXCL12 = C-X-C motif chemokine ligand 12; NOS2 = nitric oxide synthase 2; TGFB1 = transforming growth factor β 1; MMP9 = matrix metalloproteinase 9; CTSS = cathepsin S. PMA = phorbol 12-myristate 13-acetate; LPS = lipopolysaccharide; FSH = follicle stimulating hormone. (TIF) [file pone.0285607.s002.tif]

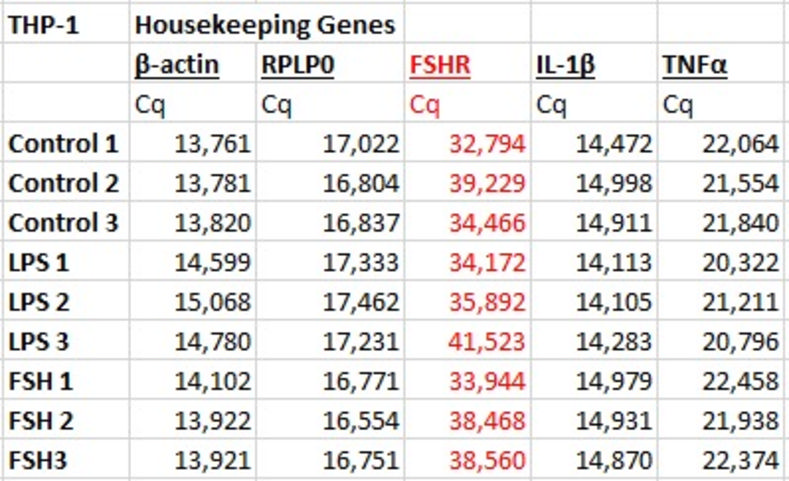

Supplement: S3 Fig — β-actin and RPLP0 were used as housekeeping genes. The data reveal that Cq cycles for FSHR were beyond our threshold of 32, while for example cytokines IL-1β and TNFα are abundantly expressed with low Cq values. (TIF) [file pone.0285607.s003.tif]

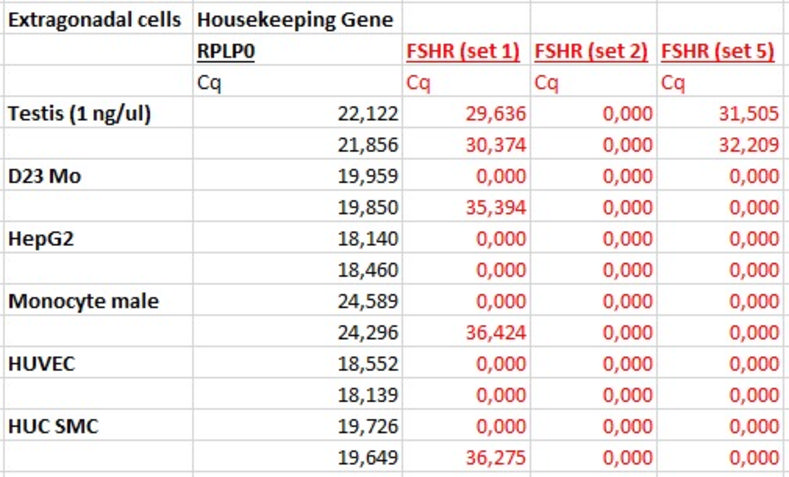

Supplement: S4 Fig — RPLP0 was used as housekeeping gene. The nomenclature of Robinson et al. was used for FSHR_set1, FSHR_set2 and FSHR_set 5. FSHR primer set 2 should only recognize the shorter FSHR variant which has been observed previously in extragonadal cells. However, here we do not see any relevant FSHR expression with any of the FSHR primer sets, except the normal FSHR variant in testis with FSHR primer set 1 (which only recognizes the normal FSHR) and set 5 (which should recognize the normal and short FSHR variant). (TIF) [file pone.0285607.s004.tif]

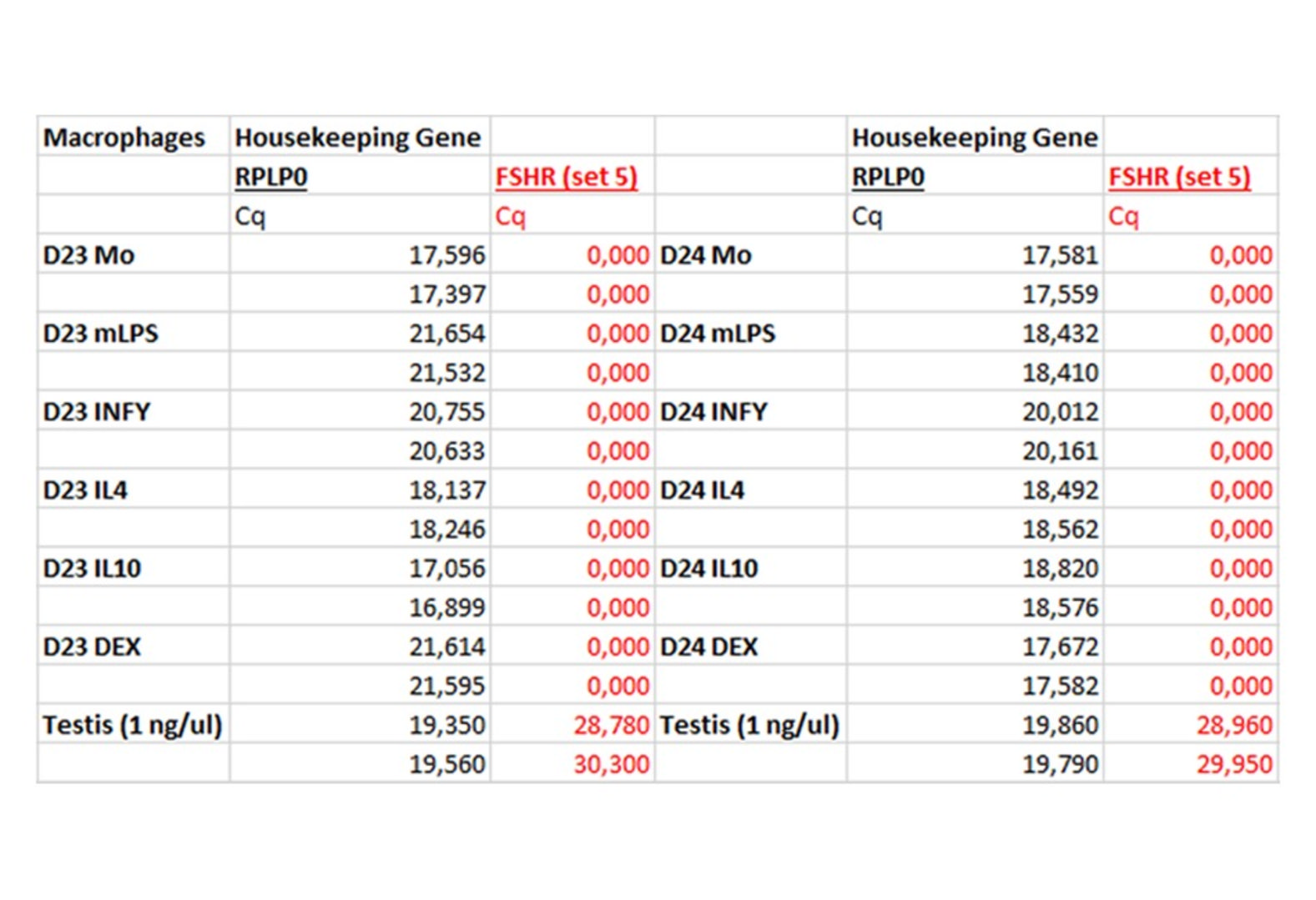

Supplement: S5 Fig — The various stimuli were control (Mo), LPS (mLPS), interferon- γ (INFY), IL-4 (IL4), IL-10 (IL10) and dexamethasone (DEX). Testis was used as positive control. RPLP0 was used as housekeeping gene. The data show that there was no amplification of FSHR transcripts in the macrophages, irrespective of being stimulated by various stimuli. These were samples from a previously published study: DOI: 10.3389/fimmu.2019.02887. (TIF) [file pone.0285607.s005.tif]

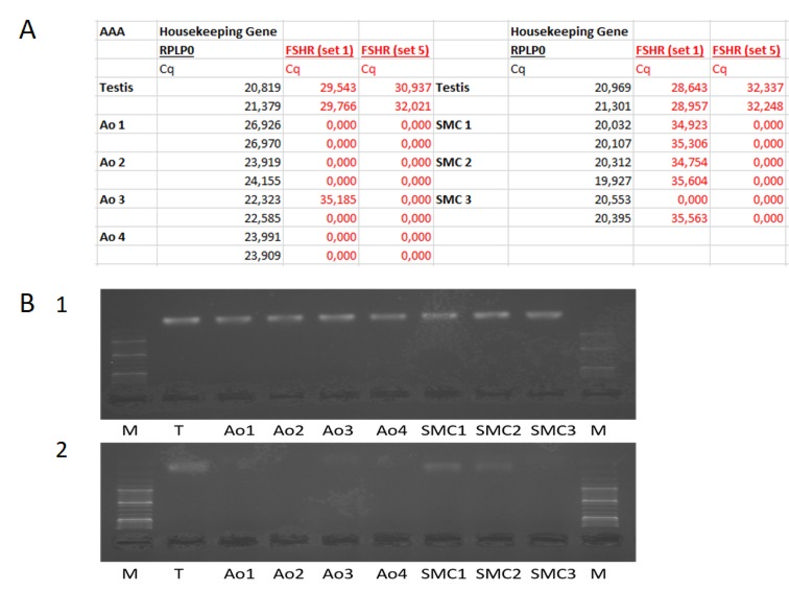

Supplement: S6 Fig — FSHR_1 stands for primer set 1 and FSHR_5 for primer set 5 (nomenclature of Robinson et al.). RPLP0 was used as housekeeping gene. The data reveal that no FSHR transcripts were detected in AAA tissue and that in AAA-SMCs the Cq was beyond our Cq threshold (A). The PCR products on gel of RPLP0 as housekeeping gene (1) and FSHR (2; primer set 1) of AAA samples and AAA-derived SMCs. M stands for marker, T for testis (positive control), Ao for the AAA samples and SMC for the AAA-derived SMCs. The data show that the housekeeping gene is detected in all samples, and that the FSHR is only detected in testis and in two AAA-SMCs (B). (TIF) [file pone.0285607.s006.tif]

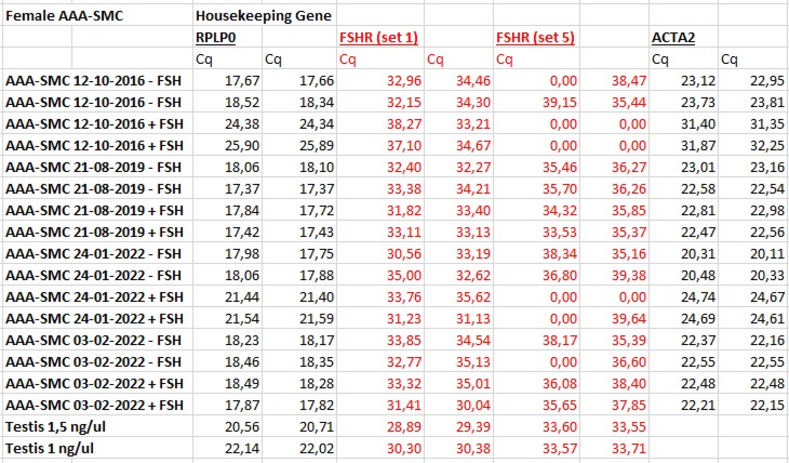

Supplement: S7 Fig — Testis RNA was used as positive control. RPLP0 was used as housekeeping gene. FSHR set 1 and FSHR set 5 correspond to the nomenclature of Robinson et al. and show high Cq values or lack of amplification for FSHR transcripts. ACTA2 was used as a typical smooth muscle cell marker with relatively high expression. (TIF) [file pone.0285607.s007.tif]

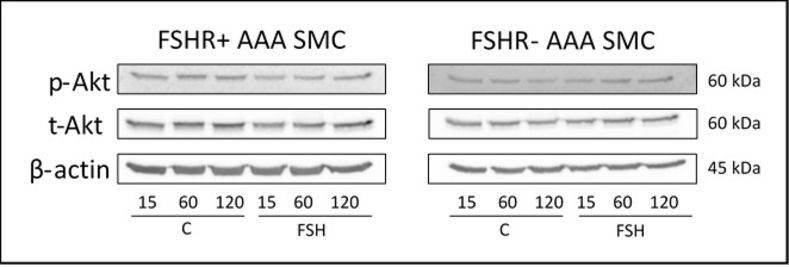

Supplement: S8 Fig — Western blot of phosphorylated AKT (p-AKT, Ser473) and total AKT (t-AKT) after FSH stimulation (FSH) versus control (C) for 15-60-120 minutes in AAA-SMC with (+) FSHR and AAA-SMC without (-) FSHR expression from postmenopausal female donors. Beta-actin was used as control. No regulation is observed in AKT phosphorylation by FSH in SMCs with or without FSHR expression. (TIF) [file pone.0285607.s008.tif]

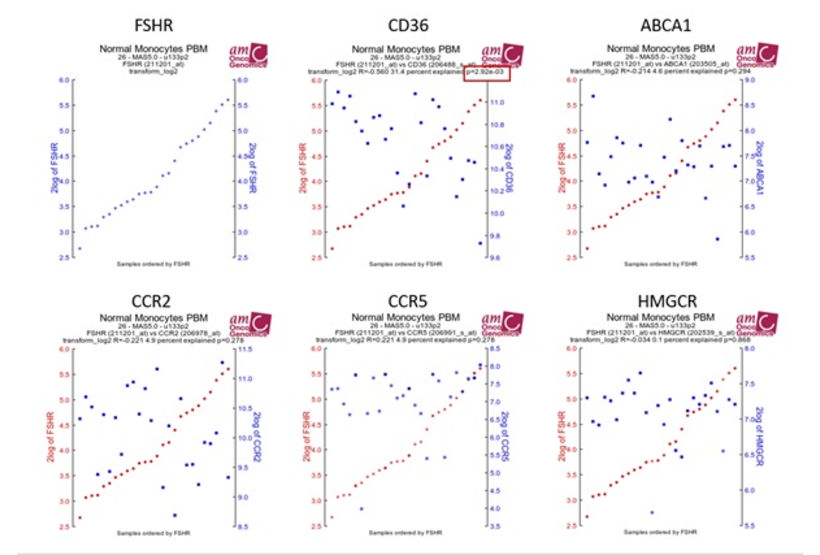

Supplement: S9 Fig — Exploration using the R2 platform (http://r2.amc.nl) for an association between FSHR and various typical monocyte markers in circulating monocytes from 26 females (age 20–45) with either high or low bone mass (GSE7158). First, there is variable FSHR expression on monocytes of these 26 females. Secondly, CD36 expression in these monocytes was significantly inversely correlated with FSHR expression. All other markers are examples that these markers did not associate with FSHR expression in monocytes. ABCA1 = ATP-binding cassette transporter-1; CCR2 = C-C motif chemokine receptor type 2 or MCP1 receptor; CCR5 = C-C motif chemokine receptor type 5 or RANTES receptor; HMGCR = 3-hydroxy-3-methylglutaryl coenzyme A (HMG-CoA) reductase. (TIF) [file pone.0285607.s009.tif]

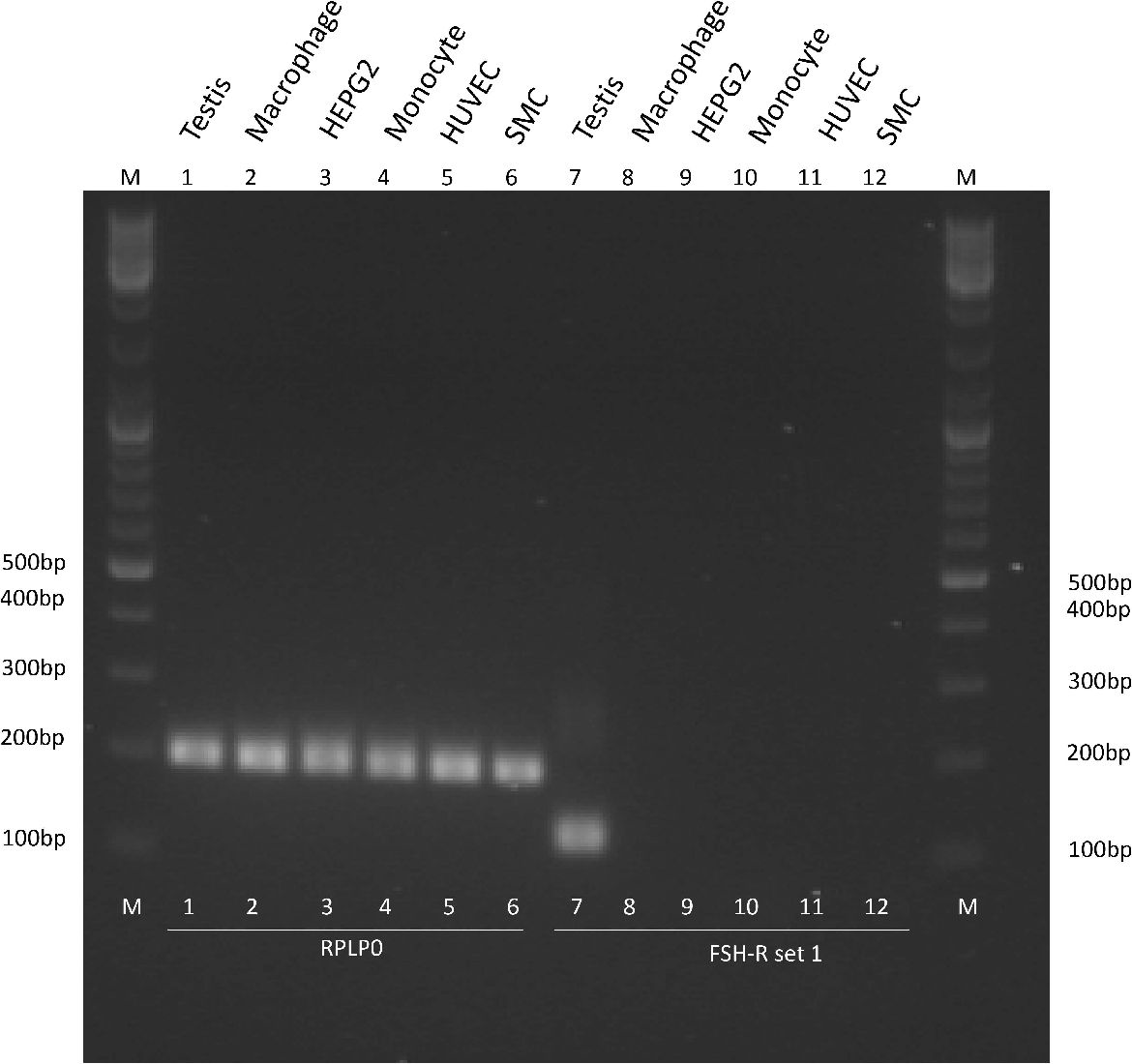

Supplement: S1 Raw images — (TIF) [file pone.0285607.s011.tif]

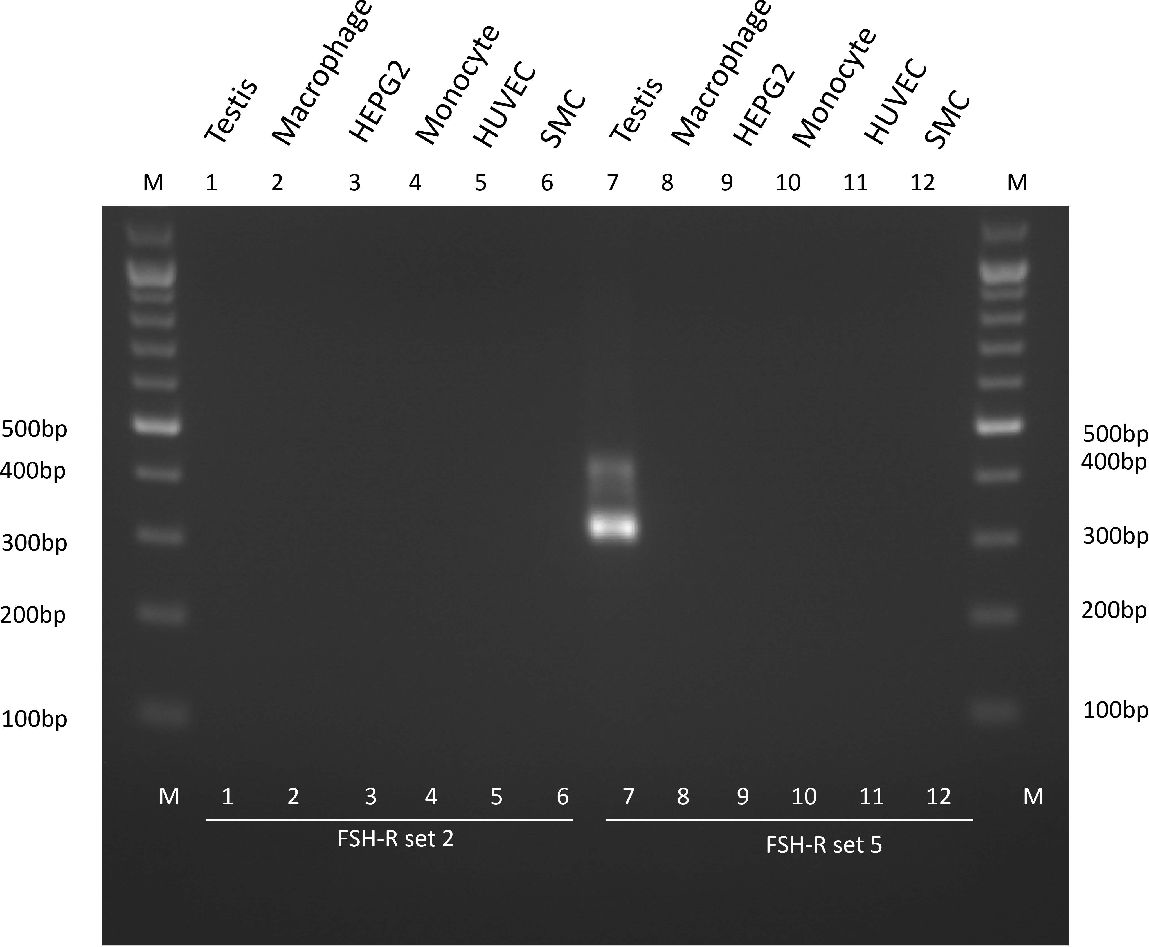

Supplement: S2 Raw images — (TIF) [file pone.0285607.s012.tif]

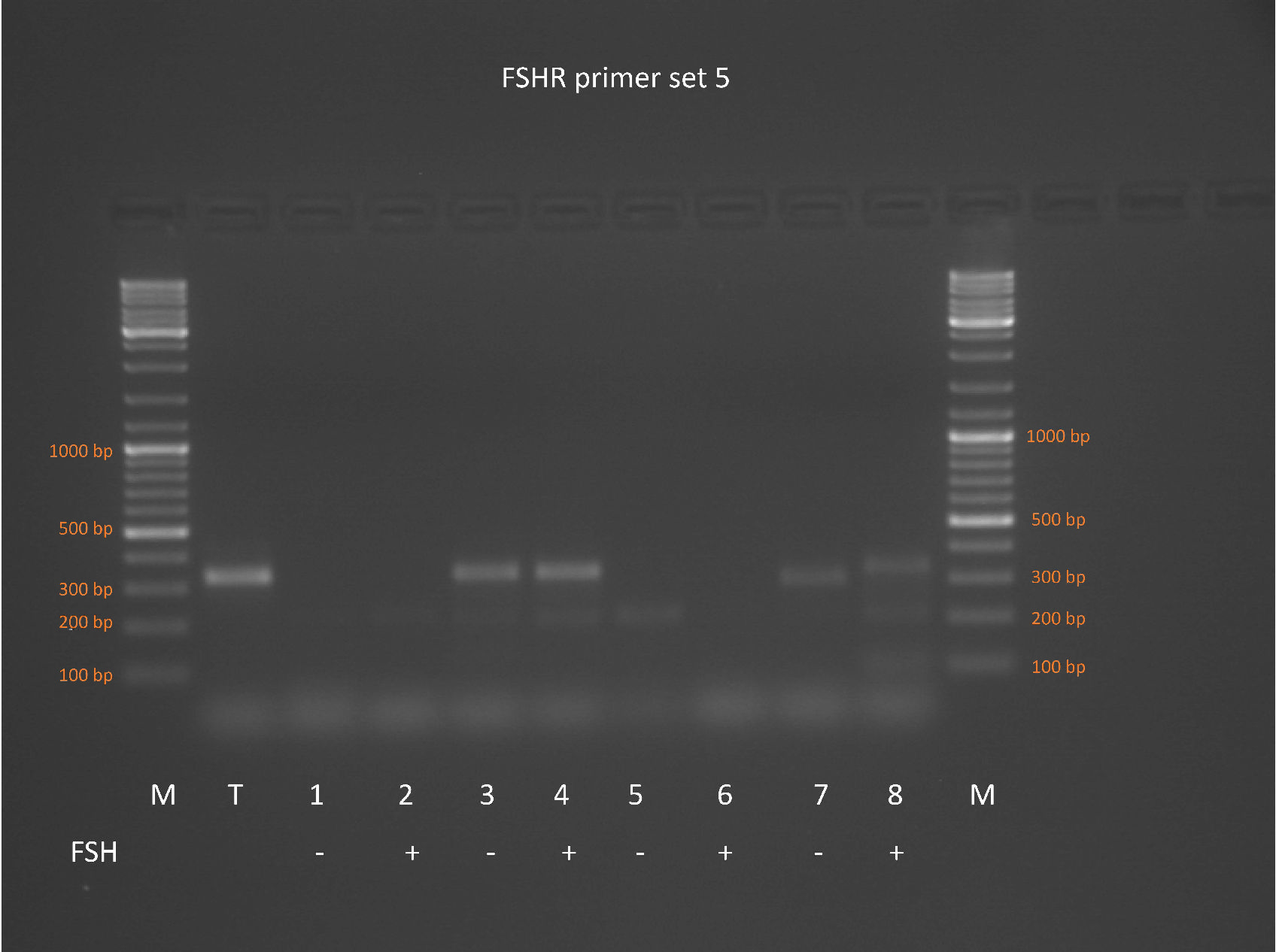

Supplement: S3 Raw images — (TIF) [file pone.0285607.s013.tif]
