# Supplementary material for: Exploring the expression and potential function of follicle stimulating hormone receptor in extragonadal cells related to abdominal aortic aneurysm
Source: PLoS One. 2023 May 25;18(5):e0285607. doi: 10.1371/journal.pone.0285607 (PMC10212101; doi:10.1371/journal.pone.0285607)
Supplement: S1 Table — (DOCX) [file pone.0285607.s010.docx]

**Supplemental Table S1.**

Human primer sequences used for real-time quantitative polymerase chain reaction.

| **Gene** | **Forward primer sequence (5’ to 3’)** | **Reverse primer sequence (5’ to 3’)** |
| --- | --- | --- |
| *GAPDH* | CCAGGGCTGCTTTTAACTCTGG | ATCGCCCCACTTGATTTTGG |
| *RPLP0* | TCGACAATGGCAGCATCTAC | ATCCGTCTCCACAGACAAGG |
| *ACTB* | GGACTTCGAGCAAGAGATGG | AGCACTGTGTTGGCGTACAG |
| *FSHR* | GGAAAAGCTTGTCGCCCTCA | AGAGAGGATCTCTGACCCCT |
| Primer set 1 (ref. 12) | CTCACCAAGCTTCGAGTCATCCAA | AAGGTTGGAGAACACATCTGCCTCT |
| Primer set 2 (ref. 12) | TGGACCAGTCATTCTCTCTGA | CTCTGCTGTAGCTGGACTCAT |
| Primer set 5 (ref. 12) | AGCCTCTGGACCAGTCATTCT | CTCTGCTGTAGCTGGACTCAT |
| *IL1B* | TGGCAGAAAGGGAACAGAAAGG | GTGAGTAGGAGAGGTGAGAGAGG |
| *IL6* | CGCCTTCGGTCCAGTTG | TCGTTCTGAAGAGGTGAGTG |
| *CXCL8* | TGTTCCACTGTGCCTTGGTTTCTCC | TGCTTCCACATGTCCTCACAACATCAC |
| *ICAM1* | CAGAGGTTGAACCCCACAGT | CCTCTGGCTTCGTCAGAATC |
| *VEGF* | CTACCTCCACCATGCCAAGTG | CACACAGGATGGCTTGAAGATG |
| *CCL2* | CCTAGCTTTCCCCAGACACC | CCCAGGGGTAGAACTGTGG |
| *ACTA2* | GACAATGGCTCTGGGCTCTGTAA | ATGCCATGTTCTATCGGGTACTT |
| *TNF* | AGGACACCATGAGCACTGAAAG | AGGAGAGGCTGAGGAACAAG |
| *CXCL12* | TCGAAAGCCATGTTGCCAGAGCC | GCCGGGCTACAATCTGAAGGGC |
| *NOS2* | AGCACATTCAGATCCCCAAG | GATTCTGCCGAGATTTGAGC |
| *TGFB1* | GGGACTATCCACCTGCAAGA | CCTCCTTGGCGTAGTAGTCG |
| *MMP9* | GCGTCGTGGTTCCAACTC | CGGTCGTCGGTGTCGTAG |
| *CTSS* | CTGGGCATGAACCACCTGGGAGA | CTCTGCCACTGGCTGGGAACTCT |
